# Butyrate Alters Pyruvate Flux and Induces Lipid Accumulation in Cultured Colonocytes

**DOI:** 10.3390/ijms222010937

**Published:** 2021-10-10

**Authors:** Anna F. Bekebrede, Thirza van Deuren, Walter J. J. Gerrits, Jaap Keijer, Vincent C. J. de Boer

**Affiliations:** 1 Human and Animal Physiology, Wageningen University and Research, 6708 WD Wageningen, The Netherlands; anna.bekebrede@wur.nl (A.F.B.); t.vandeuren@maastrichtuniversity.nl (T.v.D.); jaap.keijer@wur.nl (J.K.); 2 Animal Nutrition Group, Wageningen University and Research, 6708 WD Wageningen, The Netherlands; walter.gerrits@wur.nl

**Keywords:** butyrate, glucose oxidation, metabolite interactions, metabolic flux, short-chain fatty acids

## Abstract

Butyrate is considered the primary energy source of colonocytes and has received wide attention due to its unique health benefits. Insight into the mechanistic effects of butyrate on cellular and metabolic function relies mainly on research in in-vitro-cultured cells. However, cells in culture differ from those *in vivo* in terms of metabolic phenotype and nutrient availability. For translation, it is therefore important to understand the impact of different nutrients on the effects of butyrate. We investigated the metabolic consequences of butyrate exposure under various culturing conditions, with a focus on the interaction between butyrate and glucose. To investigate whether the effects of butyrate were different between cells with high and low mitochondrial capacity, we cultured HT29 cells under either low- (0.5 mM) or high- (25 mM) glucose conditions. Low-glucose culturing increased the mitochondrial capacity of HT29 cells compared to high-glucose (25 mM) cultured HT29 cells. Long-term exposure to butyrate did not alter mitochondrial bioenergetics, but it decreased glycolytic function, regardless of glucose availability. In addition, both high- and low-glucose-grown HT29 cells showed increased lipid droplet accumulation following long-term butyrate exposure. Acute exposure of cultured cells (HT29 and Caco-2) to butyrate increased their oxygen consumption rate (OCR). A simultaneous decrease in extracellular acidification rate (ECAR) was observed. Furthermore, in the absence of glucose, OCR did not increase in response to butyrate. These results lead us to believe that butyrate itself was not responsible for the observed increase in OCR, but, instead, butyrate stimulated pyruvate flux into mitochondria. Indeed, blocking of the mitochondrial pyruvate carrier prevented a butyrate-induced increase in oxygen consumption. Taken together, our results indicate that butyrate itself is not oxidized in cultured cells but instead alters pyruvate flux and induces lipid accumulation.

## 1. Introduction

Butyrate is a short-chain fatty acid (SCFA) that, in humans, is mostly produced through fiber fermentation by microbiota in the colon. Butyrate is mainly known as the primary source of energy for healthy colon cells [1] and has also been found to have beneficial health effects. In the colon, it is found to protect against colorectal cancer [2], and evidence from pre-clinical models shows that butyrate can be used as a treatment for intestinal bowel diseases (IBD) [3] and even diarrheal diseases [4]. Apart from local effects in the colon, butyrate was also found to affect whole-body metabolism, such as the prevention of high-fat-diet-induced obesity [5] and fatty liver disease caused by obesity and a high-fat diet [5].

Butyrate exerts its beneficial effects through various mechanisms, of which the most interesting one is by serving as a direct mitochondrial substrate. Apart from thermogenic effects in adipocytes [6] and effects on colonocyte-neighboring immune cells [7], effects of butyrate have prominently been studied in colonocytes. As an energy source in colonocytes, butyrate provides cellular energy in the form of adenosine triphosphate (ATP) [8]. Germ-free mice lack butyrate-producing bacteria, and their colonocytes were found to be energy deprived [9]. Re-administration of butyrate restored colonic energy levels, indicating that butyrate is indeed a crucial energy source [9]. Because butyrate catabolism in mitochondria utilizes oxygen for oxidation in colonocytes, it lowers oxygen levels, which prevents vascular oxygen from leaking into the anoxic colonic lumen [10]. In some diseases, such as IBD, butyrate oxidation is decreased, especially during phases of active disease [11,12,13]. The mechanistic explanation for the decrease in butyrate oxidation is likely the ongoing inflammation, which alters the ability of colonocytes to take up and metabolize butyrate [14], highlighting that butyrate metabolism is central to many of its physiological functions.

Studies that aim to identify mechanisms for how butyrate affects metabolic and cellular functions often rely on in-vitro-cultured colonic cell lines. However, the metabolic phenotype of cultured cells often differs from that of cells *in vivo* [15,16]. Cells in the physiologic context of the human body are exposed to different and changing nutrient and oxygen levels, whereas *in vitro* cultured cells are typically grown in culture medium that is rich in nutrients and oxygen. Interestingly, the *in vitro* effects of butyrate were found to be influenced by the culture medium composition, showing the importance of understanding how different nutritional factors impact the outcome of butyrate exposure [17,18]. In addition, cultured colonic cells display the Warburg effect, which means that they preferentially oxidize glucose as an energy substrate in normoxic conditions, instead of butyrate, which is typically highly available *in situ* in the colon and preferred by *in vivo* colonocytes [16]. Nutritional conditions and consequential nutrient interactions thus differ from the *in vivo* situation, which may impact the effects of butyrate exposure and therefore pose limitations to the translatability of the *in vitro* findings to *in vivo* human physiology.

Cultured cells can be steered towards oxidative metabolism, making them more comparable to the oxidative phenotype of *in vivo* colonocytes. Attempts at more physiological culturing of colonic cells have been performed extensively, by changing the medium nutrient composition [19,20,21], as well as by co-culturing with other cell types or by growing cells as spheroids or organoids. Supplying low glucose levels or replacing glucose with galactose stimulates mitochondrial metabolism because of the lower ATP yields in the glycolytic pathway [19,20,21,22]. The obtained increased mitochondrial metabolism could increase mitochondrial butyrate oxidation, but other metabolic routes of butyrate utilization have also been described. For example, butyrate was shown to be incorporated into lipids in cultured colonic cells [23]. Butyrate can also be used as a substrate for post-translational protein acylation modification, either when converted to acetyl-CoA as a source for protein acetylation or more directly via butyryl-CoA as a source for protein butyrylation [24,25].

An important remaining question is how different metabolic phenotypes, i.e., glycolytic or oxidative, and nutritional environments influence the effects of butyrate exposure. In particular, we are interested in the interaction between butyrate and glucose, since cultured cells are often cultured in media containing high levels of glucose. Interestingly, glucose was found to dictate whether butyrate was able to induce apoptosis in cultured colonocytes [26]. In addition, butyrate exposure led to increased glucose uptake in Caco-2 cells [27], but decreased glucose uptake in HT29, HCT116 and LoVo cells [28,29]. In contrast, butyrate was also found to impact the oxidation of glucose, although the direction of this effect differed between studies [30,31,32,33,34]. To better understand the fate of butyrate under different metabolic and nutritional conditions, we studied butyrate utilization in colonic cells in detail. We used a panel of colonic cell lines as well as colon-derived primary cells to demonstrate that butyrate is likely not oxidized by mitochondria but instead rewires pyruvate flux and fuels lipid droplets, even in conditions of high mitochondrial oxidative metabolism.

## 2. Results

### 2.1. Long-Term Exposure to Butyrate Decreases Glycolytic Function of Both High- and Low-Glucose-Cultured HT29 Cells

**Figure 1:**
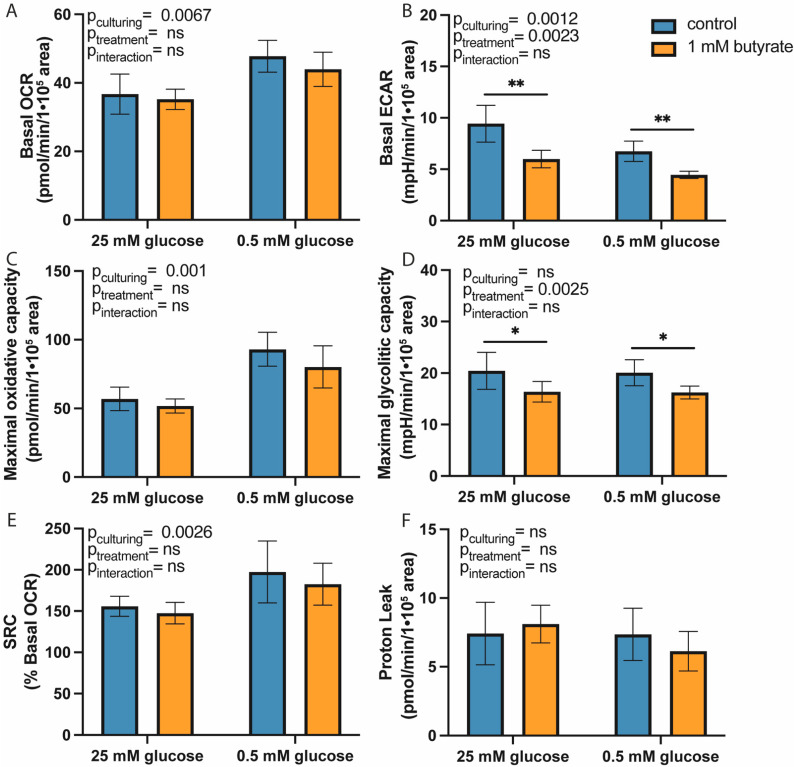
Metabolic flux analysis of high- and low-glucose-cultured HT29 cells following 72 h exposure to 1 mM butyrate. (**A**) Basal OCR. (**B**) Basal ECAR. (**C**) Maximal oxidative capacity. (**D**) Maximal glycolytic capacity. (**E**) SRC (%) and (**F**) Proton leak. (*N* = 3. Data are presented as mean ± SD; significance was determined using two-way ANOVA with factors culturing (25 mM or 0.5 mM glucose level in culture medium) and treatment (only medium or butyrate exposure), followed by Bonferroni post-hoc analysis. * = *p* < 0.05, ** = *p* < 0.01).

**Figure 2:**
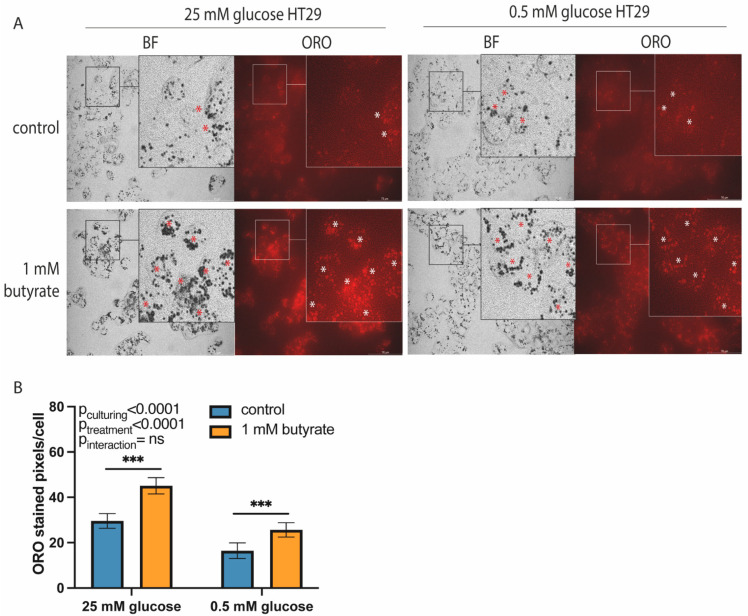
Lipid accumulation in high- and low-glucose-cultured HT29 cells following 72 h exposure to 1 mM butyrate. (**A**) Representative images showing brightfield (BF), nuclear (DAPI) and Oil-Red-O (ORO) staining in 25 mM and 0.5 mM glucose-cultured HT29 cells following 72 h exposure to 1 mM butyrate. Red and white stars (*) indicate lipid droplets stained using Oil-Red-O. (**B**) Representative bar graph showing quantification of ORO-stained image area in pixels corrected for cell number. (Images and bar graph are from a representative experiment of a total of *N* = 2. Data are presented as mean ± SD; significance was determined using two-way ANOVA followed by Bonferroni’s post-hoc analysis. *p* < 0.001 = ***). Scale bar = 70 µM.

To investigate whether the long-term effects of butyrate on mitochondrial function were affected by glucose, we cultured HT29 cells using standard high-glucose medium (25 mM) or custom low-glucose medium (0.5 mM glucose). HT29 cells cultured in 0.5 mM glucose had higher basal oxygen consumption rate (OCR; increase of 9.9 ± 2.7 pmol/min/1 × 10^5^ area, *p* = 0.0067), higher maximal oxidative capacity (increase of 32.3 ± 6.4 pmol/min/1 × 10^5^ area, *p* = 0.001), higher spare respiratory capacity (increase of 38.4 ± 14% of basal OCR, *p* = 0.0026) and lower basal extracellular acidification rate (ECAR; reduction of 2.1 mpH/min/1 × 10^5^ area, *p* = 0.0012) as compared to cells cultured in 25 mM glucose, which indicated that our low-glucose-cultured HT29 cells relied more on mitochondrial metabolism than high-glucose cultured HT29 cells (Figure 1). In addition, 0.5 mM glucose-cultured cells had lower lipid levels than 25 mM glucose-cultured HT29 cells (reduction of ±10.8 ORO-stained pixels/cell; Figure 2). Since butyrate is a short-chain fatty acid that can be converted to acetyl-CoA in the mitochondria, we expected that long-term butyrate exposure would affect mitochondrial parameters. Surprisingly, butyrate did not alter mitochondrial parameters but changed glycolytic parameters significantly. Butyrate lowered basal ECAR by 36% in 25 mM glucose-cultured HT29 cells and by 38% in 0.5 mM glucose HT29 cells (*p* = 0.0023; Figure 1B) and maximal ECAR by approximately 20% in both culture conditions (*p* = 0.0025). Although 25 mM and 0.5 mM glucose-cultured cells had significantly different mitochondrial functions, the effect of butyrate on metabolic flux was not different between these conditions. We hypothesized that instead of increased mitochondrial flux, *de novo* lipogenesis could be affected by butyrate exposure. We did not observe altered gene expression of two *de novo* lipogenesis-related genes (Supplementary Figure S1), indicating that lipogenesis upon butyrate exposure is likely not regulated on the gene expression level but instead could be regulated on the enzymatic or post-translational level. Remarkably, exposure to butyrate increased lipid accumulation to the same extent in 25 mM and 0.5 mM glucose-cultured HT29 cells (in both cases, a significant increase of approximately 150%, *p* < 0.0001; Figure 2). In the literature, it is often suggested that the pentose phosphate pathway (PPP) is increased following butyrate exposure to regenerate NADPH for lipogenesis [23,28]. However, we did not observe altered gene expression of key PPP genes after 72 h butyrate exposure, in either the 25 or the 0.5 mM glucose-cultured HT29 cells (Supplementary Figure S1). Thus, long-term exposure to butyrate reduced the glycolytic function of HT29 cells without affecting mitochondrial flux parameters. The effect of butyrate was independent of culture conditions, since there was no difference between 25 and 0.5 mM glucose-cultured HT29 cells. To ensure that our findings were not affected by altered cell viability or cell death following butyrate exposure, we investigated cell viability and cell counts in response to 1 mM butyrate exposure. Cell viability was not changed in high-glucose-cultured HT29 cells after 48 h exposure to 1 mM butyrate (Supplementary Figure S2A), and the cell count in high- and low-glucose-cultured HT29 cells was also not significantly affected by 48 h exposure to 1 mM butyrate (Supplementary Figure S2B).

### 2.2. Butyrate Acutely Lowers Glycolytic Function in Multiple Colon-Derived Cell Lines

**Figure 3:**
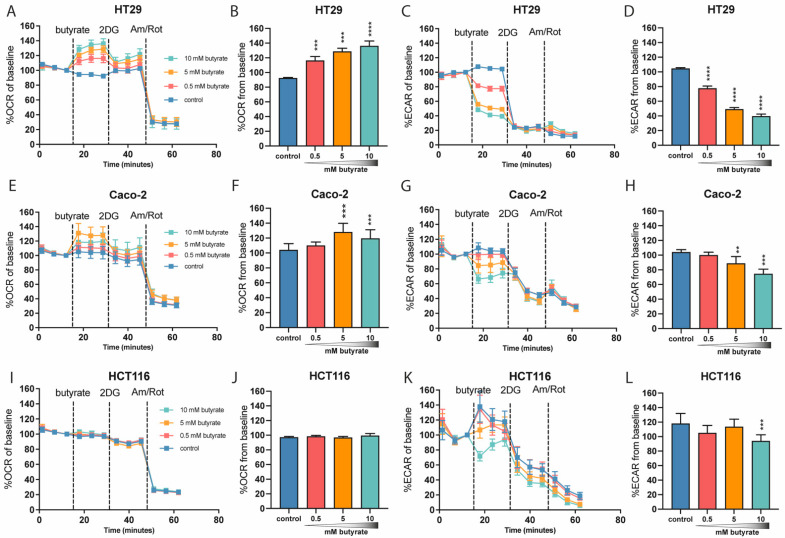
Metabolic flux analysis of multiple colon-derived cell lines following acute butyrate exposure. (**A**–**D**) 25 mM cultured HT29 cells (representative experiment of *N* = 4 independent experiments consisting of *n* = 5–6 wells). (**E**–**H**) Caco-2 cells (representative experiment of *N* = 3 independent experiments consisting of *n* = 9–15 wells). (**I**–**L**) HCT116 cells (representative experiment of *N* = 3 independent experiments consisting of *n* = 12 wells; significance was determined for one representative experiment using repeated-measures ANOVA followed by Bonferroni’s post-hoc analysis. *p* < 0.01 = **, *p* < 0.001 = ***, *p* < 0.0001 = ****).

Since we observed a decrease in glycolytic function only after longer-term butyrate exposure, we went on to investigate the acute effects of butyrate on metabolic flux. To investigate this, we exposed high-glucose-cultured HT29, Caco-2 and HCT116 cells to an acute injection of butyrate during the Seahorse Extracellular Flux analysis. The 25 mM glucose-cultured HT29 cells and Caco-2 cells responded to acute butyrate exposure with increased OCR in a dose-dependent manner (Figure 3A,B,E,F). Given that 2DG (2-deoxyglucose) blocks glycolysis, which necessitates the sole use of mitochondria for ATP generation, we expected that 2DG would further increase the butyrate-induced OCR. Strikingly, OCR was attenuated by subsequent 2DG injection (Figure 3A,B,E,F). The decrease in OCR following 2DG injection suggests that butyrate is not the only substrate responsible for the increase in OCR, and that at least part of the observed increase in oxidation rate is fueled by glucose. This notion is strengthened by the observation that acute exposure to butyrate strongly reduced ECAR in a dose-dependent manner in the 25 mM glucose-cultured HT29 and Caco-2 cell lines (Figure 3C,D,G,H). Interestingly, the HCT116 cells did not respond to the acute butyrate exposure by increasing their OCR, but there was also an inhibitory effect on ECAR, significant at the highest butyrate concentration of 10 mM (Figure 3I–L).

**Figure 4:**
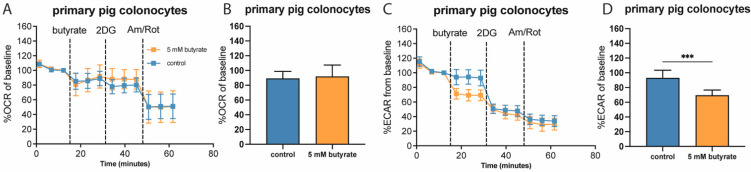
Metabolic flux analysis of primary pig colonocytes following acute butyrate exposure in the presence of 2.5 mM glucose. (**A**,**B**) Time-course and bar graph showing acute effect of control or butyrate injection and subsequent injection of 2DG and Antimycin A/Rotenone on OCR. (**C**,**D**) Time-course and bar graph showing acute effect of control or butyrate injection and subsequent injection of 2DG and Antimycin A/Rotenone on ECAR in primary pig colonocytes. (*n* = 3. Data are shown for a colonocyte isolation of one representative pig and are presented as mean ± SD for *n* = 12 wells; significance was determined using the replicates of one individual using repeated-measures ANOVA followed by Bonferroni’s post-hoc analysis. *p* < 0.001 = ***).

To verify whether the effect of butyrate on OCR and ECAR was not limited to merely cultured cell lines, we isolated primary pig colonocytes and analyzed mitochondrial and glycolytic parameters in response to an acute butyrate injection in these primary cells. Similar to HCT116 cells and different from Caco-2 and HT29 cells, primary pig colonocytes did not show any changes in OCR upon acute exposure to 5 mM of butyrate (Figure 4A,B). However, as with all cultured cell lines tested, upon injection of butyrate, pig colonocytes showed reduced glycolytic flux by approximately 50% of the total inhibition that was achieved by 2DG (Figure 4C,D). This indicates that the acute effects of butyrate on glycolysis are not restricted to cultured cells.

### 2.3. Butyrate Alters Pyruvate Flux in Cultured Colon Cell Lines

Our results suggest that at least part of the increased oxidation rate is due to the oxidation of substrates other than butyrate. Glucose-derived pyruvate seems to be the most likely substrate for two reasons. Firstly, acute butyrate exposure increased OCR, which was at least partly reversed by exposure to 2DG, as 2DG inhibits glycolysis and thus pyruvate production. This decreased pyruvate supply for mitochondrial oxidation is the most likely cause for the decrease in OCR observed upon 2DG injection. Secondly, acute butyrate exposure led to a simultaneous increase in OCR and decrease in ECAR. Altered pyruvate flux seems to be the most likely cause, since pyruvate can either enter the mitochondria, which contributes to OCR, or be converted into lactate, which contributes to ECAR.

**Figure 5:**
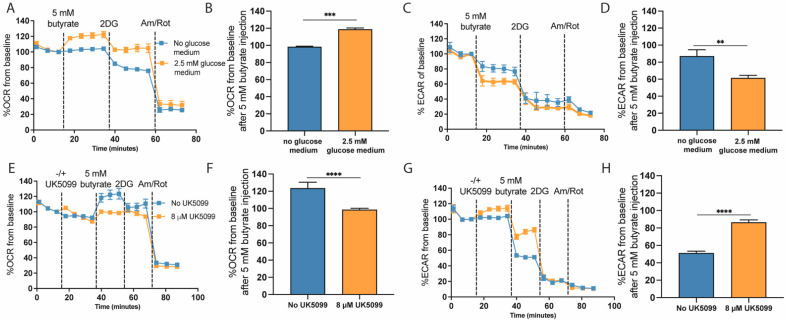
The altered response to acute butyrate exposure as a consequence of glycolysis inhibition in HT29 cells. (**A**,**B**) OCR response following butyrate exposure in absence or presence of 2.5 mM glucose in the medium (representative experiment of *N* = 2 independent experiments consisting of *n* = 6 wells). (**C**,**D**). ECAR response following butyrate exposure in absence or presence of 2.5 mM glucose in the medium (representative experiment of *N* = 2 independent experiments consisting of *n* = 6 wells). (**E**,**F**) OCR response upon inhibition of pyruvate oxidation using 8 µM UK5099 in the presence of 2.5 mM glucose (representative experiment of *N* = 3 independent experiments consisting of *n* = 13–15 wells). (**G**,**H**) ECAR response upon inhibition of pyruvate oxidation using 8 µM UK5099 (representative experiment of *N* = 3 independent experiments consisting of *n* = 13–15 wells; significance was determined for one representative experiment using repeated-measures ANOVA followed by Bonferroni’s post-hoc analysis. *p* < 0.01 = **, *p* < 0.001 = ***, *p* < 0.0001 = ****).

To test our hypothesis that glucose-derived pyruvate flux is altered by acute exposure to butyrate, we exposed 25 mM cultured HT29 cells to butyrate in the presence or absence of 2.5 mM glucose. We observed that the increase in OCR upon butyrate exposure did indeed not occur when there was no glucose present in the medium (Figure 5A,B). In addition, the decrease in ECAR upon acute butyrate exposure was less apparent when no glucose was available (Figure 5C,D). These results show that glucose is required to achieve the acute increase in OCR and decrease in ECAR upon butyrate exposure. To confirm that butyrate specifically alters pyruvate flux toward mitochondrial oxidation and away from lactate production, we inhibited the mitochondrial pyruvate carrier (MPC) with UK5099. Exposure to UK5099 induced a slight but significant (*p* < 0.0001) decrease in OCR and increase in ECAR. This confirms that UK5099 indeed inhibits MPC, since pyruvate can no longer directly enter the mitochondria and is therefore converted into lactate. When MPC was blocked with UK5099, the increased OCR (Figure 5E,F) and decreased ECAR (Figure 5G,H) following acute butyrate exposure were no longer observed. We therefore believe that the increase in OCR observed in the cultured cell lines HT29 and Caco-2, as well as the decrease in ECAR that could be seen in HCT116 and primary pig colonocytes, was not due to direct oxidation of butyrate but instead resulted from a rerouting of the pyruvate away from lactate production and towards mitochondrial oxidation.

## 3. Discussion

Although butyrate is considered the main energy source for *in vivo* colonocytes [1], it is unclear whether ex vivo and cultured colonocytes are able to oxidize butyrate. Whether or not oxidation can take place in a certain cell is an important issue with regard to the usability of these models when investigating butyrate’s translatable mechanisms of action. In this study, we have used both cultured and primary cells, as well as high- and low-glucose culturing conditions, to show that butyrate does not seem to be primarily oxidized in cultured cells but instead fuels lipid droplets and alters pyruvate flux away from lactate production and towards mitochondrial oxidation. These findings imply that the nutritional environment is an important determinant for the effects of butyrate in cultured cells. Our research shows that a better understanding of the *in vivo* nutrient composition, and how different nutritional environments may affect the impact of butyrate, is needed to further study the effects of butyrate on colonocyte metabolism.

We hypothesize that the effects that we observed on pyruvate flux and lipid accumulation are intertwined. Even though butyrate oxidation is sometimes even observed in cultured cells [35], we observed that butyrate primarily alters the flux of pyruvate towards mitochondrial oxidation. We conclude this based on four main findings: (1) increased OCR upon butyrate exposure elicits a simultaneous decrease in ECAR, indicating that less lactate is being produced; (2) the increased OCR following butyrate exposure is largely blunted by the addition of 2DG, indicating that part of the OCR is derived from glucose; (3) the absence of glucose in the medium prevented the butyrate-induced increase in OCR; (4) UK5099, which blocks pyruvate entry into mitochondria, likewise largely prevents the butyrate-induced increase in OCR. At the same time, we saw a clear increase in lipid droplets, even in the more oxidative low-glucose-cultured HT29 cells, indicating that this was not solely due to diminished mitochondrial capacity of the cells. Likely, butyrate is directly incorporated into lipids, because it does not have to be broken down completely into acetyl-CoA but can be directly elongated from butyryl-CoA [36]. As others have suggested before us, we hypothesize that mitochondrial pyruvate oxidation is increased to generate the NADPH and acetyl-CoA that are needed to convert butyryl-CoA into lipids. There are multiple pathways through which NADPH can be generated. One is through the PPP, which has been observed to be upregulated upon butyrate exposure by some [23,28,29,30], but not by us and others [29]. NADPH can also be generated through the cytosolic conversion of malate to pyruvate or citrate to oxoglutarate, which regenerate cytosolic NADPH. For this, pyruvate would need to be (partially) oxidized in the TCA cycle first, which would explain the need for the increased pyruvate oxidation observed in our experiments. Mechanistically, butyrate possibly alters pyruvate flux by post-translationally inhibiting pyruvate dehydrogenase kinase (PDK), which alleviates the inhibition of PDK on pyruvate dehydrogenase (PDH) [37]. Butyrate was also shown to inactivate SIRT3, resulting in increased acetylation and activation of PDH [38]. In this way, more pyruvate is able to be converted into acetyl-CoA and enter the TCA cycle. Butyrate was also shown to increase the expression of pyruvate kinase M2 (PKM2), which stimulates the conversion of phosphoenolpyruvate (PEP) to pyruvate [39]. Another possibility is that butyrate directly interacts with the PDH complex through butyrylation or crotonylation, but we have not yet found any clear evidence for this, neither in our own experiments nor in the literature. Although it remains unclear by which means, our data clearly indicate the upregulation of pyruvate oxidation, which is possibly driven by an increased need for NADPH.

Butyrate is already known to be incorporated into lipids [40]. Butyrate possibly contributes to *de novo* lipogenesis through a shared pool with ketone bodies that is separate from that of acetate and propionate [41]. Interestingly, the degree to which butyrate contributes to *de novo* lipogenesis seems to be largely dependent on substrate availability. The presence of glucose in the medium was found to greatly increase the amount of butyrate that is converted into lipids [40,41]. Corresponding with our findings, butyrate was found to increase lipogenesis in cultured colonocytes [23]. However, on a whole-body level, butyrate administration mainly decreased lipid accumulation in the liver [42,43]. The decreased lipid storage in the liver upon butyrate exposure could be due to increased lipolysis in hepatocytes themselves and other cells such as adipocytes [44,45,46,47], but possibly also because of increased lipid storage in other organs, such as the muscle [45] or even intestine. Nonetheless, it is unclear whether butyrate can induce lipid storage *in vivo* in the colon, and how the nutritional environment affects the outcome of butyrate exposure in the colon. In our experiments, there was no difference in lipogenesis between high- and low-glucose-cultured cells, but the low-glucose-cultured cells still had sufficient pyruvate and glutamine available to them, which could partly replace the role of glucose breakdown as a source for NADPH generation. Our experiments thus show that it is important to further investigate the impact of metabolic phenotype as well as nutritional environment to better understand the effects of butyrate.

In our experiments, it remains undetermined whether the lipids are directly derived from butyrate, or perhaps originate from other substrates. We therefore propose to perform isotope-tracing studies for future experiments to deepen our understanding of the fate of butyrate under different nutritional environments and metabolic phenotypes. Nevertheless, our experiments have already generated novel insights into the role of butyrate in regulating colonocyte metabolism. An interesting proposition for why cultured cells store butyrate as lipid droplets, instead of oxidizing this substrate, is that these cells are maintained in a nutrient-rich environment, and butyrate is an excess nutrient that can be stored for later use.

## 4. Materials and Methods

### 4.1. Cell Culture

The human colorectal (adeno)carcinoma cell lines HT29 (HTB-38), Caco-2 (HTB-37) and HCT116 (CCL-247) were originally obtained from the American Type Culture Collection (ATCC, Manassas, VA, USA). The 25 mM glucose-cultured HT29, Caco-2 and HCT116 cells were maintained in 25 mM glucose Dulbecco’s Modified Eagle’s Medium (DMEM) (42430-025, Thermo Fisher Scientific, Pittsburgh, PA, USA, 227289), supplemented with 10% *v/v* fetal bovine serum (FBS), 25 mM HEPES (15630-056, Thermo Fisher Scientific, Pittsburgh, PA, USA, 227289), 1 mM sodium pyruvate (11360-039, Thermo Fisher Scientific, Pittsburgh, PA, USA, 227289), 1% *v/v* glutamax 100× (35050-038, Thermo Fisher Scientific, Pittsburgh, PA, USA, 227289) and 1% *v/v* antibiotic–antimycotic (15240-062, Thermo Fisher Scientific, Pittsburgh, PA, USA, 227289). The 0.5 mM glucose-cultured HT29 cells were cultured in DMEM (11966-025, Thermo Fisher Scientific, Pittsburgh, PA, USA, 227289) supplemented with 0.5 mM glucose, 10% *v/v* FBS, 25 mM HEPES, 1 mM sodium pyruvate, 1% *v/v* glutamax 100× and 1% *v/v* antibiotic–antimycotic. The 0.5 mM glucose cells were passaged at least 10 times before being used for experiments. All cell cultures were grown in T75 flasks and kept in a humidified incubator at 37 °C in 95% air and 5% CO_2_. Cells were passaged or used for experiments when a confluency of 80–90% was reached.

### 4.2. Intestinal Colon Cell Isolation

Colon samples were obtained from slaughterhouse material of approximately 10-week-old piglets. A 20 cm section of mid-sigmoid colon was obtained immediately after slaughter, and intestines were placed in aerated Krebs Henseleit Buffer containing 5 mM glucose (hereafter referred to as modified-KHB, K3753, Sigma-Aldrich, St. Louis, MO, USA) supplemented with 2.5 g/L Bovine serum albumin (BSA, A7906, Sigma-Aldrich, St. Louis, MO, USA). After this, the intestines were flushed with modified-KHB. Then, they were inverted, and a sac was created using dialysis clamps by filling them with modified-KHB. The sacs were incubated for 20 min in Ca^2+^-free KHB buffer containing 20 mM EDTA and 10 mM DTT in a shaking 37 °C water bath. Following this washing step, intestines were re-verted and filled with an isolation buffer containing Ca^2+^-free KHB buffer, 2.5 g/L BSA and 400 U/mL hyaluronidase type IV (3884, Sigma-Aldrich, St. Louis, MO, USA). After a fifteen-minute incubation, the intestines were gently massaged and cells were collected, washed and counted using a Cellometer K4 (Nexcelom Bioscience, Lawrence, MA, USA), and viability was simultaneously assessed by staining with ViaStain (CS2-0106, Nexcelom Bioscience, Lawrence, MA, USA). Cells were taken up in KHB medium containing 1 mM HEPES (4-(2-Hydroxyethyl)piperazine-1-ethanesulfonic acid) and 2.5 mM glucose, pH 7.4.

### 4.3. Metabolic Flux Analysis with Seahorse XFe96 Analyzer

To investigate the long-term metabolic consequences of butyrate, both the 25 mM and 0.5 mM glucose-cultured HT29 cells were exposed to 1 mM butyrate for 72 h prior to metabolic analysis. Cells were first exposed for 48 h in a T75 flask, after which they were detached and taken up in fresh medium containing 1 mM butyrate. Then, cells were counted and seeded in a XF96 cell plate at 3 × 10^4^ cells/well (25 mM glucose-cultured cells) or 3.5 × 10^4^ (0.5 mM glucose-cultured cells). After an additional 24 h of exposure to 1 mM butyrate, medium was switched to XF DMEM assay medium supplemented with either 25 or 0.5 mM XF glucose (for the 25 and 0.5 mM glucose-cultured HT29 cells, respectively), 2 mM XF glutamine and 1 mM XF pyruvate. In addition, 1 mM sodium butyrate was added to the exposed cells. XF96 cell plates were kept in a non-CO_2_ incubator set to 37 °C for one hour prior to metabolic flux analysis.

To assess the acute metabolic consequences of butyrate, 25 mM glucose-cultured HT29, Caco-2 and HCT116 cells were plated in a XF96 cell plate at 3 × 10^4^ cells/well and left to attach overnight. One hour prior to the metabolic analysis, culture medium was replaced with bicarbonate-free KHB supplemented with 2.5 mM glucose and 1 mM HEPES, set at pH 7.4 at 37 °C. XF96 cell plates were kept in a non-CO_2_ incubator set to 37 °C for one hour prior to metabolic flux analysis.

The primary isolated colonocytes were taken up in KHB medium containing 1 mM HEPES and 2.5 mM glucose set at pH 7.4, and plated at a concentration of 9 × 10^4^ cells/well in a XF96 cell plates that were coated with Cell-Tak (354240, Corning, New York, NY, USA), according to the manufacturer’s protocol. XF96 cell plates were kept in a non-CO_2_ incubator set to 37 °C for one hour prior to metabolic flux analysis.

A Seahorse Extracellular XFe96 analyzer (Seahorse Bioscience, Agilent Technologies, Santa Clara, CA, USA) was used to assess the metabolic consequences of butyrate pre-treatment or acute exposure. To assess the metabolic consequences of 72 h exposure to butyrate, extracellular flux analyses (XF assays) were performed using serial injections of 1.5 µM Oligomycin (O4875), 1.5 µM carbonyl cyanide-p-trifluoromethoxyphenylhydrazone (FCCP; C2920), a combination of 1.25 µM Rotenone (R8875) and 2.5 µM Antimycin A (A8674), all purchased from Sigma-Aldrich. The XF assay protocol typically consisted of 12 measurement cycles of 3 min, with 2 min of mixing between measurements. To measure the acute effects of butyrate, XF assays were performed using serial injections of 0, 0.5, 5 or 10 mM sodium butyrate, 50 mM 2-deoxyglucose (2DG) and a combination of 1.25 µM Rotenone and 2.5 µM Antimycin A. To block the mitochondrial pyruvate carrier, 8 µM UK5099 (PZ0160, Sigma-Aldrich, St. Louis, MO, USA).

### 4.4. Imaging Procedure

Following the XF analysis, cells were fixed using 4% neutrally buffered formalin solution (NBF, 252549, Sigma-Aldrich, St. Louis, MO, USA). Nuclei were stained using 4′,6-diamidino-2-phenylindole (DAPI, Sigma-Aldrich, St. Louis, MO, USA), and images were taken of the middle of each well using the Cytation 1 (Nexcellom Biosciences, Lawrence, MA, USA). Images were processed in ImageJ (Win64, version 1.52, NI, USA). First, the background was subtracted. Then, the nucleus-covered area was determined by setting an automatic threshold, and the number of nucleus-covered pixels was determined. The number of pixels covered with nuclei was then used to normalize the XF assays.

### 4.5. Oil-Red-O Staining and Image Processing

To assess lipid accumulation following 72 h butyrate exposure, 0.5 mM and 25 mM glucose-cultured HT29 cells were exposed to 1 mM butyrate in a T75 flask for 48 h. Then, they were plated in tissue-culture-treated 12-well plates for an additional exposure of 24 h to 1 mM butyrate, after which they were stained with Oil-Red-O (ORO, O0625, Sigma-Aldrich, St. Louis, MO, USA). A working solution of 0.3% *v/v* ORO in 60% isopropanol was used to stain lipid droplets. First, cells were fixed using 4% NBF, followed by ORO and DAPI staining to stain lipid droplets and cell nuclei, respectively.

Images were obtained using the Leica DM8 inverted microscope, using a 40× magnification. Gain, image intensity and exposure time were kept equal for all images obtained. ImageJ was used to quantify areas covered by lipid droplets. First, the image was converted to an 8-bit image and inverted. Then, the background was subtracted using a rolling ball algorithm. The threshold was then manually set and kept the same for all images, and the selected area was measured. In addition, the DAPI images were used to count the number of nuclei, and the area covered was corrected for the number of cells in the picture.

### 4.6. WST-1 Cell Viability Assay

To assess cell viability following 48 h butyrate exposure, 25 mM glucose-cultured HT29 cells were seeded at 30,000 cells/well. Following overnight attachment, cells were exposed to only medium or 1 mM butyrate for 48 h. Then, WST-1 reagent (5015944001, Roche, Basel, Switzerland) was added according to the manufacturer’s protocol, and absorbance was read using the Synergy™ HT Multi-Detection Microplate Reader (BioTek Instruments, Inc., Winooski, VT, USA) at 450 nm. Cell viability is reported as percentage of control.

### 4.7. RNA Extraction and Semi-Quantative Real-Time Polymerase Chain Reation (qPCR)

Cells were washed with cold Hanks’ Balanced Salt Solution (HBSS) and directly scraped using RLT buffer supplemented with 1% β-mercaptoethanol. RNA was then isolated using the RNeasy mini kit (74106, Qiagen, Hilden, Germany). The quality and quantity of purified RNA was determined using a NanoDrop spectrophotometer (ND-1000). cDNA was synthesized with the iSCRIPT cDNA synthesis kit (170-8891, BioRad, Hercules, CA, USA) in the Eppendorf-Master cycler (5′ 25 °C, 30′ 42 °C, 5′ 85 °C, 10 °C ∞). Gene expression was measured using the CFX96 Touch Real-Time PCR Detection System (BioRad, Hercules, CA, USA) and SYPBR green master mix (1725006CUST, BioRad, Hercules, CA, USA). The cycling program was set as follows: 3′ 95 °C, 40 cycles of 15″ 95 °C and 45″ 60 °C, 1′ 95 °C and 1′ 65 °C, followed by melt curve analysis by increasing temperature every 10″ with increments of 0.5 °C. Primers were designed using NCBI Primer BLAST. Normalized expression was calculated according to the ΔΔCq method, by making use of multiple reference genes (rsp15 and B2M), using the CFX maestro software (BioRad, Hercules, CA, USA). An overview of the primers used can be found in Table 1.

### 4.8. Statistical Analysis and Data Representation

Data are presented as mean ± SD, unless stated otherwise. Statistical analyses and data visualizations were performed using GraphPad Prism v.9 (GraphPad Software, CA, USA). Statistical testing was performed using Student’s *t*-test and two-way or repeated-measures ANOVA, followed by Bonferroni’s post-hoc analysis when appropriate and as stated in the figure legends. A *p*-value of <0.05 was considered statistically significant.

## 5. Conclusions

Butyrate is not oxidized to a great extent in high- and low-glucose-cultured HT29 cells, but instead accumulates in lipid droplets. Acute exposure to butyrate directly increases the OCR of various cultured cells; however, the increase in OCR observed is not caused by butyrate oxidation but reflects increased pyruvate oxidation. These findings are important for interpreting the role of butyrate in regulating colonic metabolism in health and disease.

## Figures and Tables

**Table 1:** Details of primers.

Symbol	RefSeq	Forward Primer *	Reverse Primer *	bp
*RSP15*	NM_003194.5	AGAAGCCGGAAGTGGTGAAGAC	AGAGGGATGAAGCGGGAGGAG	220
*B2M*	NM_004048.4	TGCCGTGTGAACCATGTG	GCGGCATCTTCAAACCTC	92
*ACLY*	NM_001096.3	GGACTTCGGCAGAGGTAGAG	TGATCAGCTGGTCTGGCTTG	227
*ACACA*	NM_198834.3	GGGCTAGGTCTTTTTGGAAGTG	GGCCAAGGGAGATGGTTCAT	104
*G6PD*	NM_001360016.2	AAGCGCAGACAGCGTCAT	TGAAGGTGTTTTCGGGCAGA	215
*PGD*	NM_002631.4	TGTGACTGGGTGGGAGATGA	TCCCTGATCTTTGGCAGCAG	257

* from 5′ to 3′, bp = fragment length.

## Data Availability

The data presented in this study are available upon request.

## Supplementary Materials

The following are available online at https://www.mdpi.com/article/10.3390/ijms222010937/s1, Figure S1: Relative gene expression of key pentose phosphate pathway genes in long-term butyrate-exposed high- and low-glucose HT29 cells; Figure S2: Viability and cell count of HT29 cells after 48 h exposure to 1 mM butyrate.
