# Supplementary material for: Butyrate Alters Pyruvate Flux and Induces Lipid Accumulation in Cultured Colonocytes
Source: Int J Mol Sci. 2021 Oct 10;22(20):10937. doi: 10.3390/ijms222010937 (PMC8539916; doi:10.3390/ijms222010937)
Supplement: Supplementary file 1 [file ijms-22-10937-s001.zip › ijms-1405231-supplementary.pdf]

Supplementary figures

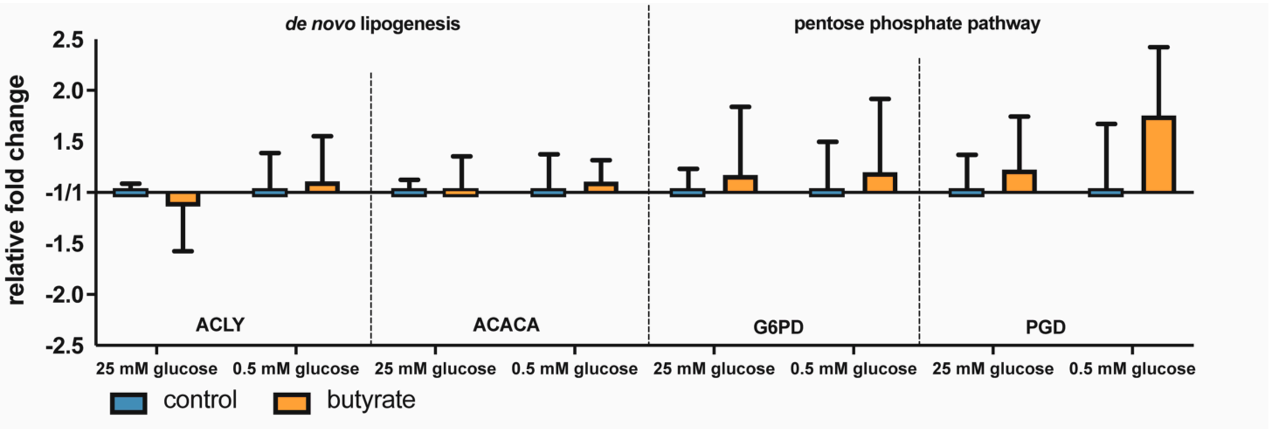

Supplementary Figure S1. Relative gene expression of *de novo* lipogenesis and pentose phosphate pathway genes in long-term butyrate exposed high and low glucose HT29 cells. Relative gene expression of ATP citrate lyase (ACLY), acetyl-Coenzyme A carboxylase alpha (ACACA) glucose-6-phosphate dehydrogenase (G6PD) and 6-phosphoglconate Dehydrogenase (PGD) in 25 mM and 0.5 mM glucose cultured HT29 cells exposed to only medium or 1 mM butyrate for 72 hours. (Data are derived from N=3-4 independent experiments and are presented as mean ± SD; significance was determined using two-way ANOVA followed by Bonferroni's post-hoc analysis).

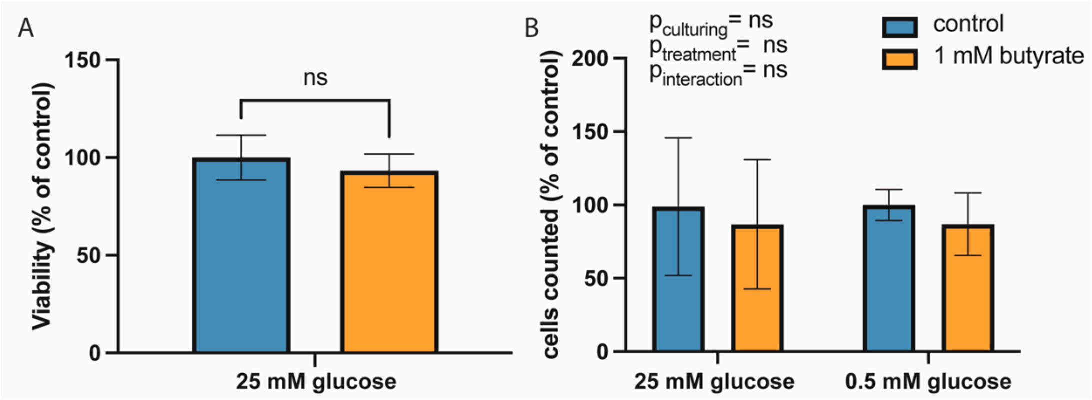

Supplementary Figure S2. Viability and cell count after 48 hours exposure to 1 mM butyrate. A. Viability of high glucose (25 mM) cultured HT29 cells after 48 hours exposure to 1 mM butyrate (N=2) B. Cell-count of high (25 mM) and low glucose (0.5 mM) cultured HT29 cells after 48 hours exposure to 1 mM butyrate (N=3; data are presented as mean ± SD; significance was determined using Student's t-test (A) or two-way ANOVA followed by Bonferroni's post-hoc analysis (B)).
